# Nine Years of Continuous Flow LVAD (HeartMate 3): Survival and LVAD-Related Complications before and after Hospital Discharge

**DOI:** 10.3390/jcdd11100301

**Published:** 2024-09-30

**Authors:** Rodrigo Sandoval Boburg, Spiros Lukas Marinos, Michael Baumgaertner, Christian Jörg Rustenbach, Christoph Salewski, Isabelle Doll, Rafal Berger, Christian Schlensak, Medhat Radwan

**Affiliations:** Department of Thoracic and Cardiovascular Surgery, Tübingen University Hospital, 72076 Tübingen, Germany; rodrigo.sandoval-boburg@med.uni-tuebingen.de (R.S.B.); michael.baumgaertner@med.uni-tuebingen.de (M.B.); christian.rustenbach@med.uni-tuebingen.de (C.J.R.); isabelle.doll@med.uni-tuebingen.de (I.D.); rafal.berger@med.uni-tuebingen.de (R.B.); christian.schlensak@med.uni-tuebingen.de (C.S.); medhat.radwan@med.uni-tuebingen.de (M.R.)

**Keywords:** left ventricular assist device, HeartMate 3, end-stage heart failure

## Abstract

Background: End-stage heart failure is associated with high mortality. Recent developments such as the left ventricular assist device (LVAD) have improved patient outcomes. The HeartMate 3 LVAD is a novel centrifugal pump that was developed to provide hemodynamic support in heart failure patients, either as a bridge-to-transplant (BTT), myocardial recovery, or destination therapy (DT). Our objective was to evaluate the survival rates and LVAD-related complications of the HeartMate 3 LVAD before and after hospital discharge in our center. Methods: We retrospectively reviewed all patients implanted with the HeartMate 3 LVAD in our institute between September 2015 and June 2024. Patients who received a Heart Ware Ventricular Assist Device (HVAD) and HeartMate 2 LVAD devices were excluded. The primary endpoint was survival before and after hospital discharge. The secondary endpoints included an incidence of serious LVAD adverse events (bleeding, major infection, hemolysis, device thrombosis and malfunction, and neurological dysfunction) and the causes of re-admission along the follow-up period. Results: A total of 48 consecutive HeartMate 3 LVAD patients were enrolled in this study. The mean age was 56.1 ± 10.6 years. A total of 72.9% of patients received LVAD therapy as a BTT, 14.6% as DT, 10.4% as a bridge-to-decision, and 2.1% as a bridge-to-recovery. A total of 85.4% of patients were discharged after implantation. The main cause for in-hospital mortality was right ventricular failure (8.3%), followed by stroke, abdominal bleeding, and multi-organ failure (2.1% each). One patient (2.1%) had successful heart transplantation, 26 patients (63.4%) are still on LVAD support, and 11 (26.8%) patients have died during follow-up. The main cause of mortality after hospital discharge was sepsis, which occurred in 9.8% of patients, followed by right ventricular failure, non-LVAD-related causes, unknown causes with two (4.9%) cases each, and one case of fatal stroke (2.4%). During the follow-up, there was no need for LVAD replacement. Conclusions: HeartMate 3 LVAD is associated with excellent in-hospital survival rates in patients with end-stage heart failure. Right ventricular failure was the main cause of death before hospital discharge, whereas sepsis was the main cause of death after hospital discharge.

## 1. Introduction

End-stage heart failure is associated with high mortality. In recent decades, developments such as left ventricular assist devices (LVAD) have improved patient outcomes, especially for those suffering from left ventricular heart failure [1]. Improved outcomes and increased durability and applicability of LVAD devices have settled this treatment as an effective option for patients with end-stage heart failure who are not suitable or who are waiting for a transplant [1,2]. Moreover, donor organ shortage caused a growing interest in LVAD not only as a bridge-to-transplant (BTT) but also as a destination therapy (DT) [3].

First-generation devices provided a pulsatile flow; the second-generation devices replaced the pulsatile flow with an axial pump and continuous flow system, which had an impeller in the blood passageway; the third-generation devices replaced the axial flow pump with a centrifugal pump and magnetically suspended rotors in the pump chamber [2,4,5,6,7]. Some of the problems of the first-generation devices were the lack of durability due to device malfunction, together with low biocompatibility. Additionally, due to the size of the devices, it was difficult for patients to carry them around; the second-generation devices caused excessive trauma to the blood, causing a great number of postoperative complications [2,4,5,6,7,8,9,10,11,12]. This problem was addressed in the last generation of devices with fewer hemocompatibility events, including pump thrombosis, being reported [8,13,14]. However, despite the improvements made in the pump designs, which ultimately led to fewer adverse events, patients are not free from complications, including infections, sepsis, right heart failure, and device malfunction [6,7,8,13,14,15].

In this study, we evaluated the survival rate, LVAD-related complications, and causes of hospital re-admission of patients with HeartMate 3 LVAD devices after hospital discharge in our center in the last 9 years.

## 2. Materials and Methods

We retrospectively analyzed 48 consecutive patients who underwent LVAD implantation with a third-generation device (HeartMate 3, Abbott, Chicago, IL, USA) in our center between September 2015 and June 2024. We collected preoperative data from the patients, such as gender, age at implantation, and indication for LVAD implantation. Echocardiographic right ventricular function was evaluated using TAPSE (trans-annular plane systolic excursion) and eyeballing preoperatively.

Postoperative data were also collected, such as the length of mechanical ventilation, length of stay (LOS) at the intensive care unit (ICU), need for an extra mechanical circulatory support device, infection, tracheotomy, hospital LOS, and in-hospital mortality.

If a temporary right ventricular assist device (RVAD) was needed intraoperatively, the standard implantation method in our center consisted of arterial cannulation of the pulmonary trunk through an 8 mm dacron prosthesis, which was tunneled under the xiphoid through the skin to make a thorax closure possible. The venous cannula was implanted percutaneously through the femoral vein and connected to an ECMO. The flow rate was adjusted according to the hemodynamic and echocardiographic evaluations.

A follow-up was performed in our LVAD outpatient clinic. The patients were seen on a regular basis every 3–6 months. If a patient was admitted to the hospital, the diagnosis was recorded. Mortality was recorded in all cases.

### 2.1. Endpoints

The primary endpoint was in-hospital and long-term survival after HeartMate 3 implantation. The secondary endpoints included LVAD-related complications, including bleeding, major infection, device thrombosis and malfunction, stroke, and causes of hospital re-admission. Adverse events were defined according to the latest ISHLT definition of adverse events for trials and registries of mechanical circulatory support [16].

### 2.2. Statistical Analyses

Statistical analyses were performed using the SSPS 23.0 (IBM Corporation, Armonk, NY, USA) software. Normal distribution was checked using the Kolmogorov–Smirnov test. The continuous variables are reported as means and standard deviation if they fulfill the criteria of a normal distribution; otherwise, the median and interquartile ranges are reported. 

The ethics committee of the University of Tübingen approved this study with the project no. 194/2020BO2. Due to the retrospective nature of this study, informed consent was not necessary.

## 3. Results

Forty of the patients in this cohort were men (83.3%), with a mean age of 56.1 ± 10.6 years at implantation. The most common indication for LVAD implantation was ischemic cardiomyopathy in 25 patients (52.1%). Thirty-five patients underwent LVAD implantation as a bridge-to-transplantation (72.9%), five as bridge-to-decision (10.4%), one as bridge-to-recovery (2.1%), and seven as destination therapy (14.6%). At the time of the LVAD implantation, most patients were an INTERMACS (Interagency Registry for Mechanically Assisted Circulatory Support) 1 profile, followed by profile 4. Regarding pre-implantation right ventricular function, 68.7% of the patients had a normal or mildly impaired function. The mean TAPSE value was 16.4 mm ± 4.7. The results are shown in Table 1.

Perioperatively, 28 (58.3%) patients developed an acute kidney injury during their stay at the ICU, 23 (47.9%) patients suffered from an infection, mainly pneumonia, 5 (10.4%) patients suffered a stroke, and 5 (10.4%) patients suffered from gastrointestinal bleeding. Postoperative bleeding was a common complication, and 19 (39.6%) patients in our cohort underwent a re-thoracotomy for this reason. Six (12.5%) patients required a temporary right ventricular assist device (RVAD) after surgery. The mean LOS at the ICU was 27.21 ± 22.7 days. The mean LOS at the hospital was 50 ± 36.1 days. Patients who suffered from postoperative acute kidney injury (AKI) had a strong trend to longer in-hospital stays compared to those without an AKI (*p*-value = 0.07).

In-hospital mortality occurred in seven cases (14.6%). The main cause for in-hospital mortality was right ventricular failure, which occurred in four patients (8.3%), followed by cerebrovascular stroke, abdominal bleeding, and multi-organ failure, with one (2.1%) patient dying of each cause. The results are shown in Table 2.

During follow-up, there was a mortality rate of 26.8% (11 patients). The main cause of mortality after hospital discharge was sepsis in four patients (9.8%), followed by late right ventricular failure in two patients (4.9%), non-LVAD related causes in two patients (4.9%), one patient died of liver carcinoma and the other of pancreatic carcinoma, an unknown cause was registered for two patients (4.9%), and one patient died of a cerebrovascular stroke (2.4%). The overall mortality rate of the cohort was 37.5%. The results are shown in Table 3.

Hospital re-admissions were caused mainly by driveline infections in four patients (9.8%), followed by bleeding complications in three patients (7.3%). Two (4.9%) of the patients who suffered a driveline infection underwent a surgical relocation; the other patients were treated conservatively. One patient (2.4%) suffered spontaneous thoracic bleeding, another had gastrointestinal bleeding, and one had postoperative bleeding after surgical debridement of a driveline infection. None of these complications were lethal. Results are seen in Table 4.

During follow-up, there were no thrombosis events reported. One (2.4%) patient had a heart transplantation, and twenty-six (63.4%) were still on LVAD support. During follow-up, there was no need for a LVAD replacement. The mean follow-up time was 1.263 ± 852 days. The recorded 1-, 2-, and 5-year survival rates were 83%, 78%, and 65%.

## 4. Discussion

The continuous flow LVAD (HeartMate 3) is associated with an excellent in-hospital survival rate in patients with end-stage heart failure, and 85.4% of the patients were successfully discharged. This correlates with larger studies in which the short-term survival was around 90% [5,7].

In this retrospective, single-center study, we analyzed the short- and long-term complications that patients suffered after implantation of a third-generation LVAD. Our patient cohort was composed mainly of males; the most common indication for LVAD implantation was ischemic cardiomyopathy, and the therapeutic objective was bridge-to-transplant in most patients. The majority of patients in this cohort were on inotropic support or mechanical circulatory support without the possibility of weaning at the time of implantation (INTERMACS profiles 1–3). The preoperative right ventricular function, as assessed by echocardiography, was normal or mildly impaired in most patients.

The demographic parameters and indications for LVAD implantation correlate with the current literature [1,2,17,18]. Despite the technological advances and improvements in LVAD devices, the complication and mortality rates of patients remain elevated. The main cause of mortality in our cohort was right ventricular failure. Right ventricular function is of paramount importance for the success of LVAD therapy, which furthermore leads to a patient’s survival [18,19].

After turning on the LVAD, the blood flow is changed, and a dilated left ventricle will become smaller. The LVAD not only changes the geometry of the LV, affecting the interventricular interdependence, but also increases the RV preload while at the same time reducing the pulmonary artery pressure and decreasing the RV afterload [19]. The preoperative right ventricular function was acceptable in most patients in our cohort; however, postoperatively, six (12.5%) of them developed a right ventricular failure. Of these patients preoperatively, two (4.2%) had a severely impaired right ventricular function, two (4.2%) had a moderate right ventricular function, one (2.1%) had a mildly impaired right ventricular function, and one (2.1%) had a normal right ventricular function. All these patients had a temporary right ventricular assist device (RVAD), and in all but one patient, the RVAD could be weaned due to improvement in right ventricular function. During follow-up, two (4.9%) patients developed late right ventricular failure and subsequently died.

Another factor that has been linked to unfavorable outcomes in LVAD patients is the pre- and postoperative renal function [15]. In this cohort, 44 (91.7%) patients had some degree of preoperative renal insufficiency. After further analyzing the data, we were able to identify that patients who needed perioperative RVAD support and patients who died perioperatively had lower preoperative glomerular filtration rates (GFR) and higher blood urea nitrogen values (BUN) compared to the patients who did not have renal insufficiency. These findings correlate with the literature [15]. Postoperative AKI was also strongly associated with a longer in-hospital stay; interestingly, the need for perioperative RVAD was not.

Our cohort suffered seven (14.5%) in-hospital deaths after implantation, and of the four (8.3%) patients who died due to right ventricular failure, three (6.2%) had a moderately impaired preoperative right ventricular function, and one (2.1%) had a mildly impaired function.

One of the major problems of the second-generation devices was related to bleeding or thrombotic complications. Due to the contact of blood with the pump, the laminar flow, and the need for anticoagulation medications, patients develop an acquired coagulopathy, which, in some cases, may be fatal [2,20]. Reports comparing the second- and third-generation LVADs have shown that the third-generation devices have significantly less pump thrombosis; reports like the MOMENTUM 3 trial suggest that higher rates of device malfunction may lead to higher rates of thrombotic complications. This is of utter importance because the rate of LVAD replacement decreased substantially, resulting in less of a burden for the patients who did not have to undergo major surgery [5,6,7,14]. Additionally, reports have shown that with the third-generation devices, the rates of bleeding and stroke complications were also lower compared to the previous generations [5,6,7,14]. None of the patients in our cohort suffered from a pump thrombosis that needed to be treated surgically or medically. Only a total of three patients suffered fatal complications; one perished due to uncontrollable abdominal bleeding, and two patients suffered a fatal stroke—one of these occurred shortly after the LVAD implantation. Our data supports the literature in that the number of bleeding complications is low in this patient cohort [15,16].

According to the literature, the most common adverse event in patients with third-generation LVADs is infection; studies have shown that up to 56% of these patients suffer from some type of LVAD-related infection [14,21]. These numbers differ from those in our cohort, with only around 18% of the patients suffering from any type of important LVAD-related infection. We believe this may be due to the strict control protocols in our LVAD outpatient clinic, which ensures that patients visit the hospital for regular check-ups every 2–3 months. Despite the low rate of infections in this cohort, 9.8% of patients died of sepsis. Our values correlate with those of other groups [14,21].

Unfortunately, due to the low number of heart transplantations conducted in Germany each year, most of the patients in this cohort are still waiting for a transplant.

### Limitations

The retrospective and single-center nature of this study is its major limitation. In order to better understand and evaluate the outcomes of patients with HeartMate 3 LVADs, bigger, multicenter prospective studies are necessary.

## 5. Conclusions

HeartMate 3 is associated with excellent in-hospital survival rates in patients with end-stage heart failure, and 85.4% of the patients were successfully discharged. Our cohort suffered no thrombosis-related complications, and there was also no need for reoperation for a LVAD replacement during follow-up. Right ventricular failure was the main cause of death before hospital discharge, whereas sepsis was the main cause of death after hospital discharge.

## Figures and Tables

**Table 1:** Patient demographics, indications, and therapy objectives.

Parameter	N = 48
Gender (m)	40 (83.3%)
Age (y)	56.1 ± 10.6
Indication for LVAD Implantation	
Ischemic cardiomyopathy	25 (52.1%)
Dilatative cardiomyopathy	19 (39.6%)
Myocarditis	4 (8.3%)
Preoperative INTERMACS Profiles	
1	16 (33.3%)
2	7 (14.6%)
3	9 (18.8%)
4	16 (33.3%)
Preoperative Echocardiographic Right Ventricular Function	
Normal	18 (37.5%)
Mildly impaired	15 (31.2%)
Moderately impaired	12 (25%)
Severely impaired	3 (6.2%)
Therapy Objective	
Bridge-to-transplant	35 (72.9%)
Bridge-to-decision	5 (10.4%)
Bridge-to-recovery	1 (2.1%)
Destination therapy	7 (14.6%)

INTERMACS: Interagency Registry for Mechanically Assisted Circulatory Support.

**Table 2:** Perioperative complications.

Complication	N = 48
Acute kidney injury	28 (58.3%)
Infection	23 (47.9%)
Gastrointestinal bleeding	5 (10.4%)
Stroke	5 (10.4%)
Re-thoracotomy	19 (39.6%)
Death	7 (14.6%)
RVAD implantation due to RV dysfunction	6 (12.5%)
Length of stay at ICU (d)	27.21 ± 22.7
Hospital length of stay (d)	50 ± 36.1

**Table 3:** Mortality.

**In-Hospital Mortality**	
**Cause**	**N = 48**
Right ventricular failure	4 (8.3%)
Stroke	1 (2.1%)
Abdominal bleeding	1 (2.1%)
Multi-organ failure	1 (2.1%)
**Out-of-Hospital Mortality**	
**Cause**	**N = 41**
Sepsis	4 (9.8%)
Right ventricular failure	2 (4.9%)
Non-LVAD related	2 (4.9%)
Unknown cause	2 (4.9%)
Stroke	1 (2.4%)

**Table 4:** Causes of hospital re-admission.

Hospital Re-Admissions	
Cause	N = 41
Driveline infection	4 (9.8%)
Bleeding complications	3 (7.3%)
Heart transplantation	1 (2.4%)
Pump thrombosis	0
LVAD replacement	0
Patients still on LVAD support	26 (63.4%)

## Data Availability

Data are contained within the article.
